# Free-Living Amoebae in Soil Samples from Santiago Island, Cape Verde

**DOI:** 10.3390/microorganisms9071460

**Published:** 2021-07-07

**Authors:** Djeniffer Sousa-Ramos, María Reyes-Batlle, Natália K. Bellini, Rubén L. Rodríguez-Expósito, José E. Piñero, Jacob Lorenzo-Morales

**Affiliations:** 1 Instituto Universitario de Enfermedades Tropicales y Salud Pública de Canarias (IUETSPC), Universidad de La Laguna (ULL), Avda. Astrofísico Fco. Sánchez s/n, 38203 San Cristóbal de La Laguna, Tenerife, Spain; djrlov.sousa@gmail.com (D.S.-R.); nkbellini@gmail.com (N.K.B.); rrodrige@ull.edu.es (R.L.R.-E.); 2 Red de Investigación Cooperativa en Enfermedades Tropicales (RICET), Universidad de Salamanca, 37008 Salamanca, Spain; 3 Departamento de Obstetricia y Ginecología, Pediatría, Medicina Preventiva y Salud Pública, Toxicología, Medicina Legal y Forense y Parasitología, Universidad de La Laguna (ULL), 38200 San Cristóbal de La Laguna, Tenerife, Spain; 4 Instituto de Física de São Carlos, Universidade de São Paulo, Caixa Postal 369, São Carlos 13560-590, SP, Brazil

**Keywords:** Cape Verde, soil, free-living amoebae, *Acanthamoeba*, Santiago island

## Abstract

Free-Living Amoebae (FLA) are widely distributed protozoa, which contain some groups considered as pathogenic microorganisms. These members are able to produce several opportunistic diseases including epithelial disorders, such as keratitis and fatal encephalitis. Even though they have been reported in numerous sources, such as soils, dust and water, there is no legislation related to the presence of these protozoa in soil-related environments worldwide. Therefore, there are no established prevention or disinfection protocols to advise the population regarding FLA infections or eliminate these microorganisms from human-related environments to date. *Acanthamoeba* spp. are the most common FLA isolated in soil samples, which is also the most common genera found in clinical cases. Thus, the aim of the present study was to evaluate the presence of potentially pathogenic FLA in human-related soil samples of Santiago Island, Cabo Verde. A total of 26 soil samples were seeded in non-nutrient agar plates (2%), incubated at 26 °C, and monitored daily to evaluate the presence of FLA. DNA was extracted from those plates on which there was suspected FLA growth, and PCR amplification of the 18S rRNA gene was carried out. A total of 17 from the 26 analysed samples were positive for FLA, where *Acanthamoeba* is the most abundant isolated genus (14/17; 82.4%), with the T4 genotype being the most common (13/14; 92.9%), followed by the T5 genotype, *A. lenticulata* (1/14; 7.1%). Moreover, *Vermamoeba vermiformis*, *Stenamoeba dejonckheerei* and *Vannella pentlandi* were isolated in three other samples. To the best of our knowledge, this is the first report of FLA presence in Cape Verde and the first report of *V. vermiformis* in beach sand worldwide.

## 1. Introduction

Free-Living Amoeba (FLA) are ubiquitous protozoa reported in several different sources, such as soils, water, dust or air [1], which contribute to the microbiological population of the environment [2]. FLAs are a polyphyletic group, with stocks arising from different branches of the protozoal ancestral tree [1]. Amoebae are among the earliest eukaryotes, which have been studied since the discovery of the early microscope [3].

*Acanthamoeba* spp., *N. fowleri*, *B. mandrillaris*, *S. pedata*, *Vahlkampfia* spp., *Paravahlkampfia* spp. [1] and *Vermamoeba* spp. [4] have been described as FLAs which are able to produce several opportunistic diseases including epithelial disorders, such as keratitis, or fatal encephalitis. These pathogenic microorganisms depend on two phases in their life cycle: a vegetative and physiologically active trophozoite and a resistant phase called cyst [3]. However, some FLA genera possess a flagellated stage, such as *N. fowleri* [1].

The main FLA diet includes microorganisms such as fungi, protozoa and bacteria, as well as organic particles [5,6]. FLAs have been shown to act as reservoirs and transmission vectors for pathogenic bacteria which are capable of living within trophozoites or even cysts [7,8]. Several bacteria species have acquired resistance mechanisms to the FLA digestive enzymes (Amoeba-Resistant Bacteria (ARB)), using these protozoa as vehicles. Moreover, the cyst stage can favour the intracellular survival of bacteria, avoiding common water disinfection systems, but are non-effective against FLA cysts [7]. The high mortality of the FLA caused encephalitis and inefficacy of the current treatments and have created the need to increase knowledge of the pathogenic protozoa. Therefore, it is necessary to investigate their environmental niches and their ability to colonize human-related environments.

Cape Verde is an Atlantic archipelago country, located about 600 to 850 kilometres (320 to 460 nautical miles) west of Cap-Vert, situated at the westernmost point of continental Africa [9]. It consists of nine volcanic islands with a combined land area of about 4033 square kilometres (1557 sq mi), and forms part of the Macaronesia ecoregion, along with the Azores, the Canary Islands, Madeira, and the Savage Isles [10]. According to the National Institute of Statistics of Cape Verde (INE 2016), Santiago Island recorded the second highest population density of Cape Verde (301 inhabitants/Km^2^). The geographical location of Cape Verde archipelago is an arid and semi-arid, hot and dry climate, with scarce rainfall and an average annual temperature of 25 °C [10]. Cape Verde has few natural resources and only five of the nine main islands (Santiago, Santo Antão, São Nicolau, Fogo, and Brava) normally support significant agricultural production [11]. On the other hand, the number of tourists increased from approximately 45,000 in 1997 to more than 115,000 in 2001 and to over 765,000 in 2018, according to the Cape Verdean statistics bureau [12]. Therefore, in the present work, we aimed to evaluate the presence of pathogenic free-living amoeba in soil samples related to human activity in Santiago Island.

## 2. Materials and Methods

### 2.1. Sample Sites and Culture of FLA

**Figure 1:**
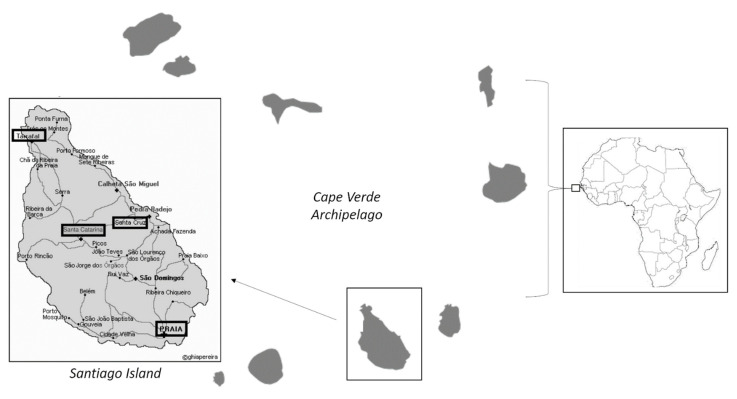
Geographical localization of the Cape Verde Archipelago and Santiago Island. Adapted from [17].

A total of 26 soil samples were collected from different towns across Santiago Island in Cape Verde (15°04′40″ N 23°37′29″ O) (Figure 1). The evaluated soils corresponded to beach sand (5/26), garden soil (6/26) and crops soil (15/26). All samples were collected directly from the land using 15 mL sterile tubes in 2019. Samples were kept at 4 °C until further processing in the laboratory. The soil was placed directly into 2% of Non-Nutrient Agar (NNA) plates and incubated at 26 °C, then monitored them daily to evaluate the presence of FLA. Plates with suspected FLA growth, following the morphological features using the Page key [13], were cloned by dilution in NNA until a monoxenic culture was obtained [14,15,16].

### 2.2. DNA Extraction

In order to extract DNA from positive samples, 1–2 mL of amoebic culture suspensions were directly placed into a Maxwell^®^ 16 tissue DNA purification kit sample cartridge (Promega, Madrid, Spain) following the manufacturer’s instructions, as has been described previously [18]. Amoebic genomic DNA yield and purity were determined using the DS-11 Spectro-photometer (DeNovix^®^, Wilmington, NC, USA).

### 2.3. PCR and Molecular Characterization of Isolates

PCR amplification of the 18S rRNA gene from the extracted DNA, was carried out using two universal primers for FLA: FLA-f 5′- CGCGGTAATTCCAGCTCCAATAGC -3′/FLA-r 5′- CAGGTTAAGGTCTCGTTCGTTAAC -3′ [19] (Tm = 62 °C) and Ame-f977 5′-GATYAGATACCGTCGTAGTC-3′ and Ame-r1534 5′-TCTAAGRGCATCACAGACCTG-3′ [20] (Tm = 55 °C), and for *Acanthamoeba* genus JDP-1f 5′- GGCCCAGATCGTTTACCGTGAA -3′ and JDP-2r 3′- TCTCACAAGCTGCTAGGGAGTCA -5′ [21] (Tm = 50 °C).

For FLA universal primers (FLA and Ame), PCRs’ amplification reactions were performed in a 50 μL mixture, containing 80 ng DNA and the 1U from the VWR Taq DNA Polymerase with 10x Key Buffer (15 mM MgCl_2_) kit. The PCR reactions were performed in 40 cycles with denaturation (95 °C, 30 s), annealing (FLAf/r 55 °C and Amef/r 62 °C, 30 s) and primer extension (72 °C, 30 s). However, in the case of *Acanthamoeba* spp. primers, the 50 μL PCR mixture contains 40 ng of DNA yield and the PCRs were performed in 35 cycles with denaturation (95 °C, 30 s), annealing (50 °C, 30 s) and primer extension (72 °C, 30 s). After the last cycles, the primer extension was maintained for 7 min at 72 °C. The expected amplicon length varies, at 500bp for JDP and Ame primers and 800bp for FLA primers. Amplification products from all PCRs were analyzed by electrophoresis through a 2% agarose gel and positive PCR products were sequenced using Macrogen Spain service (Avda. Sur del Aeropuerto, Madrid, Spain). Species identification was based on sequence homology analysis by comparison with the available DNA sequences in the Genbank database.

### 2.4. Phylogenetic Analyses

Mega X software program [22,23] was used for sequence alignment. The evolutionary history was inferred using the Neighbor-Joining method [24]. The evolutionary distances were computed using the Maximum Composite Likelihood method [22], and are shown in the units of the number of base substitutions per site. The analysis involved 23 nucleotide sequences: 15 sample sequences and 8 Genebank standard sequences. All ambiguous positions were removed for each sequence pair.

## 3. Results

From the total of 26 samples, 17 were positive for FLA growth in NNA and PCR (65.4%) (Table 1). *Acanthamoeba* spp. were the most abundantly isolated species, with a total of 14 samples (14/17; 82.4%), with the T4 genotype being the most common (13/14; 92.9%) followed by T5 genotype, *A. lenticulata* (CVS22) (1/14; 7.1%). *Vermamoeba vermiformis*, *Stenamoeba dejonckheerei* and *Vannella pentlandi* were isolated in samples of CVS3, CVS9, CVS10 respectively, with a 5.9% prevalence for each of them in our study (1/17).

**Figure 2:**
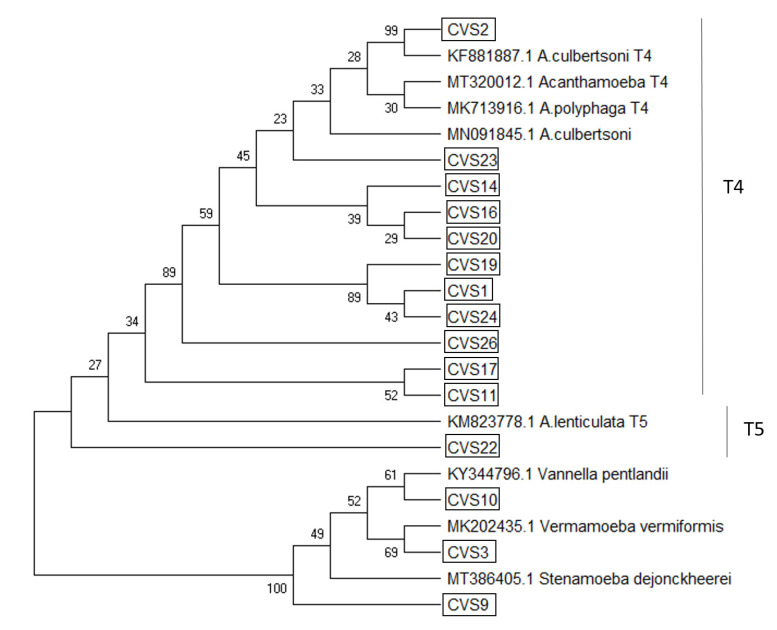
Original tree representing the evolutionary relationships of taxa carried out by the Neighbor-Joining method [22]. Phylogenetic relationship of the Amoebozoa strains isolated in the present study. The percentage of replicate trees, in which the associated taxa clustered together in the bootstrap test, are shown next to the branches [13]. The isolates obtained in the present study are identified in boxes.

The total FLA sequences obtained in the present report were deposited in the Genbank database under the accession numbers MT319991-MT320014 and presented ˃92% homology with the available DNA sequences in this database (Table 1). The evolutionary history was inferred using the Neighbor-Joining method [22]. The optimal tree for an isolated FLA phylogenetic relationship with the sum of branch length = 4.21659526 is shown in Figure 2. The isolates obtained in the present study are identified in boxes. The percentage of replicate trees in which the associated taxa clustered together in the bootstrap test (1000 replicates) are shown next to the branches [25]. The trees are drawn to scale, with branch lengths in the same units as those of the evolutionary distances used to infer the phylogenetic tree. There were a total of 2231 positions in the final dataset. Evolutionary analyses were conducted in MEGA X [23].

## 4. Discussion

In the present study, we evaluated FLA prevalence in human-related environmental soils, such as beach sand, gardens or crop soils. These microorganisms are ubiquitous environmental protists, which have contributed enormously to the microbiological contamination of water sources [2]. The lack of effective antimicrobial therapy to treat amoebic infections and the difficultly diagnosing them makes FLA infections a cause for concern. Since many cases occur in immunocompromised hosts, it is also likely that large numbers of cases escape detection, particularly in developing countries of Africa and Southeast Asia, where HIV/AIDS remains at epidemic levels [1]. Cabo Verde belongs to the Macaronesia ecoregion, along with the Azores, the Canary Islands, Madeira, and the Savage Isles [10], but this archipelago is geographically part of Africa. FLAs are pathogenic protozoa which do not require a host organism to survive [5]. Therefore, over the last 40 years, they have been considered as an emerging group of opportunistic pathogens [7]. The presence of these pathogenic protozoa was only reported in the Macaronesia region in environmental [14,15,16,18,26,27,28,29,30,31,32,33,34] and clinical veterinary samples [35,36,37] of the Canary Islands. However, there has been no report of FLA in other Macaronesia regions to date. In the current study, we detected the presence of FLA in human-related soils of Santiago Island, Cabo Verde, with a relative presence of 65.4%, forming the total analyzed samples. The authors decided to use the rRNA 18s gene as target for molecular amplification due to these rRNA transcripts being indicative of ribosomes, not just ribosomal genes. Therefore, they are likely to be derived from metabolically active cells and can be considered as markers for living microorganisms [38].

The positive samples belong to human-related environments, such as garden soils, beach sand or crop spoils. Regarding the human use of these contexts, FLA forming these contaminated soils could easily enter the human body through the nose or eyes, which are the most common path of infection [7]. Interestingly, 82.4% of the positives belong to the *Acanthamoeba* genus, which is the most pathogenic and most common FLA group [4,7]. To date, 22 *Acanthamoeba* genotypes (T1-T22) have been described [39,40], with the T4 genotype being the most frequently identified in human infection cases [41] and environmental samples [42,43]. Our findings corroborated these previous reports. The most common pathology produced by *Acanthamoeba* spp. is the sight-threatening *Acanthamoeba* keratitis (AK), followed by the more-than-95%-mortal Granulomatous Amoebic Encephalitis (GAE) [4,7]. Moreover, in the crop soil sample CV22, we demonstrated the presence of *Acanthamoeba* spp. genotype T5. Despite genotype T5 still being categorized as only potentially pathogenic [44], there have been reports demonstrating its potential pathogenicity through its weak binding capacity to corneal cells in vitro [45], a cornea infection in the USA [46], and a case of disseminated cutaneous infection [47].

On the other hand, *Vermamoeba vermiformis*, *Stenamoeba dejonckheerei* and *Vannella pentlandi* were isolated in the present research work. The pathogenic potential of *V. vermiformis* is not only determined by its ability to produce an infection by itself [3,48,49], but also by its numerous reported relationships with pathogenic bacteria [50]. Recently, *V. vermiformis* has been considered as one of the most prevalent free-living amoebae, and it has been reported as a ubiquitous and thermotolerant amoeba [51]. This amoeba was reported as the causative agent of a painful ulcer adjacent to the eye and in a case of amoebic keratitis [52,53]. However, this is the first report of *V. vermiformis* in beach sand.

As with many other FLA, *Vannella* are commonly found in the human water supply [54,55], domestic appliances [56] or crops [57]. The cyst-producing soil amoeba *Vannella pentlandii* belongs to the sub-group within the genus, presently composed of *V. placida*, *V. epipetala* and *V.fimicola* (the PEF group) [58]. To date, there has been no indication of pathogenicity of *Vannella* spp. However, it can facilitate the growth of bacteria [50,59] including *Legionella* [60] or other human pathogenic organisms [61].

The genus *Stenamoeba* was first defined in 2007 [62], where it was demonstrated that the previously denoted taxa *Platyamoeba stenopodia* [13] was not correctly placed within the Vannellida, but was a member of the Thecamoebida [62,63]. *Stenamoeba* genus were isolated from animals, soil and water environments, such as drinking water plants, sediments from a rice field, or composting facilities, in several countries (Russia, Switzerland, Denmark, Canada, The Netherlands, Germany, Tibet and Tanzania) [42]. Although the presence of *Stenamoeba* spp. have been related to fresh-water reservoirs at 37 °C [64,65], to date, no pathogenicity has been associated with this genus [65]. Finally, in 2020, a previously undescribed species *Stenamoeba dejonckheerei* was reported by Borquez-Román and colleagues [66].

## 5. Conclusions

As *Acanthamoeba* was found in most of the isolates, the need for Cape Verde authorities to further study the presence of these parasites in human-related environments is enhanced. On the other hand, *V. vermiformis* and *V. pentlandi* represent a human health risk, which is not only determined by the ability to produce an infection by itself, in the case of the first one, but also by the capacity to harbour intracellular pathogenic bacteria for both genera. Moreover, to the best of our knowledge, this is the first report of potentially pathogenic FLA in Cape Verde.

## Figures and Tables

**Table 1:** Report of the FLA species isolated from environmental soils of Cape Verde (NNA: FLA growth in non-nutrient agar culture; PCR: FLA detection by PCR;bp: base pairs; * Homology (%) related to NCBI Data Base sequence).

Sample Code	Locality	Soil Type	NNA	PCR	Isolate Specie	Seq Length (bp)	Identity (%) *	Sequence ID BLASTn
CVS1	Varzea, Praia	Garden	+	+	*Acanthamoeba* sp. T4	412	>99%	MN153014.1
CVS2	Santa Cruz	Garden	+	+	*Acanthamoeba* sp. T4	368	>92%	MN239986.1
CVS3	Camboinha, Praia	Beach	+	+	*Vermamoeba vermiformis*	422	>99%	MK202435.1
CVS9	Praia	Garden	+	+	*Stenamoeba dejonckheerei*	200	>96%	MT386405.1
CVS10	Palmarejo, Praia	Garden	+	+	*Vannella pentlandi*	189	>95%	KY344796.1
CVS11	Palmarejo, Praia	Garden	+	+	*Acanthamoeba* sp. T4	276	>95%	MT613705.1
CVS14	Santa Cruz	Crop	+	+	*Acanthamoeba* sp. T4	414	>95%	KU936118.1
CVS16	Santa Cruz	Crop	+	+	*Acanthamoeba* sp. T4	412	100%	LC373015.1
CVS17	Santa Cruz	Crop	+	+	*Acanthamoeba* sp. T4	246	>95%	MK390856.1
CVS19	Santa Cruz	Crop	+	+	*Acanthamoeba* sp. T4	403	>99%	JQ418500.1
CVS20	Santa Cruz	Crop	+	+	*Acanthamoeba* sp. T4	406	>99%	MN700306.1
CVS21	Santa Cruz	Crop	+	+	*Acanthamoeba* sp. T4	437	>98%	GU808328.1
CVS22	Santa Cruz	Crop	+	+	*Acanthamoeba lenticulata* T5	363	>99%	KM189378.1
CVS23	Santa Cruz	Crop	+	+	*Acanthamoeba* sp. T4	427	>98%	MT645313.1
CVS24	Santa Cruz	Crop	+	+	*Acanthamoeba* sp. T4	406	100%	JF702882.1
CVS25	Santa Cruz	Crop	+	+	*Acanthamoeba* sp. T4	408	>95%	KY827390.1
CVS26	Santa Cruz	Crop	+	+	*Acanthamoeba* sp. T4	315	100%	MT613705.1

## Data Availability

Not applicable.
